# Recognition, legitimacy, and the persistence of hegemonic masculinity: a relational-constitutional account of masculine reproduction

**DOI:** 10.3389/fsoc.2026.1899564

**Published:** 2026-08-03

**Authors:** Joseph L. Kidwell

**Affiliations:** Independent Researcher, Willmar, MN, United States

**Keywords:** citizenship, gender and nation, hegemonic masculinity, masculinity, militarism, nationhood

## Abstract

Dominant masculine configurations persist not through intrinsic psychological stability or widespread conscious endorsement, but through coupled recognitional and reinforcement dynamics distributed across institutional, economic, national, and digital fields. Drawing on social ontology and the sociology of recognition, this article proposes a persistence mechanics for hegemonic masculinity under contemporary conditions of labor precarity, militarism, nationalist mobilization, and digital communication. One consequence runs against intuition. Configurations are routinely reproduced by participants who do not approve of them, and routinely critiqued by those who continue to reproduce them—because participation and approval are different modes of relating to a configuration and operate in different fields. Recognition-dependent configurations stabilize through convergent field reinforcement and embodied disposition. When they rigidify, they generate characteristic harms: humiliation, exclusion, recursive alienation. They reproduce through four distinct infrastructural logics—compensatory, sacrificial, reanchoring, and amplifying—each sustaining the configuration from a different angle. What is widely described as a “crisis of masculinity” is more precisely recognitional destabilization: the erosion of the infrastructure sustaining particular hegemonic configurations, not the collapse of masculinity as such. Restorationist politics, manosphere amplification, and resurgent nationalist masculine projects are predictable consequences of that destabilization—not opposed conditions but the same dynamic operating in different registers. The questions this motivates are empirical: how do states, economies, and digital platforms recruit, reward, and marginalize masculine configurations, and under what conditions might alternative infrastructures emerge?

## 1 Introduction

The relationship between masculinity and the nation-state is being remade under contemporary conditions in ways that sociology has only begun to specify. Wars and humanitarian crises, authoritarian populism, contested borders, debates over military service, the politicization of “traditional values,” precarious labor markets, transnational migration, climate disruption, and digital cultures all shape what it means to be a man and a national subject. Two diagnoses dominate discussion of these changes: “masculinity in crisis” and “resurgent nationalism.” Both capture something real. Yet both often describe outcomes more effectively than they explain them. They name patterns of change without fully accounting for the mechanisms through which those patterns emerge, persist, and reorganize. What is needed is a more precise account of how masculinity and nationhood are reproduced, contested, and destabilized across the institutions, economies, and media environments of contemporary societies.

The sociology of masculinity already provides important resources for that task. Connell's (2005) concept of hegemonic masculinity, refined by Connell and Messerschmidt (2005), remains the field's central way of understanding dominant masculine forms as relational, contested, and historically variable rather than fixed types. Demetriou's (2001) concept of the hegemonic bloc explains how dominant forms adapt by absorbing selected elements from subordinate ones. The gender-and-nation literature developed by Nagel (1998), Enloe (1989), and Yuval-Davis (1997) shows that masculine forms are co-produced with the institutions of the nation-state. More recent work on aggrieved entitlement (Kimmel, 2013) and manosphere masculinities (Ging, 2019) extends these concerns into the contexts of labor precarity and digital communication. What remains less developed is a unified account of persistence. How do dominant masculine forms survive contestation? Why do they often intensify during periods of destabilization? Why do they disappear unevenly across social settings? And how do they reappear when displaced from one field into another?

The central claim can be stated simply. Dominant masculine configurations persist through coupled recognitional and reinforcement dynamics distributed across institutional, economic, national, and digital fields. Psychological commitment and conscious endorsement matter, but neither adequately explains persistence on its own. The argument developed here focuses instead on the social conditions that keep particular forms operative over time. Existing accounts explain dominance (Connell, 2005), adaptive hybridization (Demetriou, 2001), and the co-production of masculinity and nationhood (Nagel, 1998; Yuval-Davis, 1997). What they do not yet specify, taken individually, is a persistence mechanics—the recognitional and reinforcement dynamics through which configurations survive contestation, destabilize asymmetrically across fields, and reconstitute through partial infrastructures rather than simple reversion. That is the gap this article addresses. Recognition-dependent configurations stabilize through converging patterns of reinforcement and embodied disposition. Recognitional infrastructures reproduce them through distinct mechanisms. What is commonly described as a “crisis of masculinity” is more accurately understood as recognitional destabilization affecting particular hegemonic configurations rather than masculinity as such.

Several clarifications follow from this scope. The argument operates at the level of social mechanism rather than individual psychology or normative ideology. It explains how configurations persist; it does not offer a moral evaluation of any particular configuration. Nor does it treat recognitional dynamics as the sole source of masculine reproduction. Material conditions, economic structures, coercive institutions, and political power retain independent causal force throughout the analysis. The rigid configuration often described in public discourse through the contested label toxic masculinity is treated here as one historically specific subtype of a broader phenomenon, not as the primary object of analysis. The goal is not to construct a closed theoretical system but to develop concepts that can guide empirical investigation.

The article proceeds as follows. Section 2 situates the argument within the masculinity-and-nation literature and identifies the gap it addresses. Section 3 develops the ontology that underpins the persistence claim. Section 4 examines stabilization through habitus, reinforcement, and threshold dynamics. Section 5 introduces Lawson's account of social positioning and develops the argument about harm. Section 6 applies the framework to four contemporary infrastructures of masculine reproduction. Sections 7 and 8 consider the article's diagnostic and normative implications. Section 9 clarifies the framework's scope and limits. Section 10 concludes.

## 2 Masculinity and social reproduction: from hegemony to persistence

For nearly four decades, the sociology of masculinity has been organized around Connell's concept of hegemonic masculinity. In its original formulation (Connell, 2005), hegemonic masculinity is neither a personality type nor a collection of traits. It is a position within a structured field of gender relations: the configuration of practice that most successfully legitimates patriarchal arrangements at a particular historical moment. Other masculinitie—complicit, subordinated, and marginalized—are defined in relation to it. Three features of the concept have proven especially durable. Hegemony is relational. Its standing depends on its relationship to other masculinities and to femininities, not on any fixed content. Hegemony is contested. Dominant forms face continual pressure from subordinate and marginalized alternatives. And hegemony is historically variable. What counts as dominant changes across societies and across time. These strengths made the concept indispensable. They also left open an important question: how does a contested position remain dominant?

Connell and Messerschmidt's (2005) reformulation sharpened that question. Responding to accumulated criticism, they rejected two readings that had become increasingly common: treating hegemonic masculinity as a fixed type and treating it as a model of straightforward social reproduction. Dominant forms do not simply replicate themselves unchanged. They develop across local, regional, and global scales. They are shaped by embodiment, power, contradiction, and struggle. Subordinate masculinities influence dominant ones even while remaining subordinate. The implication is important. Persistence cannot be understood as smooth reproduction. Whatever maintains dominance must operate through contested and uneven processes.

Demetriou's (2001) concept of the hegemonic bloc provides the most influential answer from within the masculinity literature itself. Dominant forms remain dominant, in part, because they adapt. Practices associated with subordinate, marginalized, or even feminine-coded identities can be selectively incorporated into the dominant arrangement and reworked in ways that preserve its broader position. Change therefore does not necessarily signal transformation. Accommodation can become a strategy of survival. Subsequent work on hybrid masculinities extends this insight, showing that selective incorporation may leave underlying inequalities intact even while the visible content of masculinity changes.

A parallel literature places masculinity within the institutions of the nation-state. Nagel's (1998) account of nationalism and masculinity remains the clearest statement of the argument that manhood and nationhood are historically co-produced. National projects rely on gendered symbols, gendered political roles, and gendered understandings of citizenship. Enloe's (1989, 2014) work extends that analysis into militarization, borders, bases, and conscription, while highlighting the recurring importance of masculinized memory, humiliation, and hope. Yuval-Davis (1997) shows how citizenship and belonging themselves are gendered, positioning women as biological, cultural, and symbolic reproducers of the nation and men as its defenders and political agents. Together, these works establish that the nation-state is not simply a setting in which masculine forms operate. It is one of the institutions through which they are produced, stabilized, and reasserted.

Recent scholarship has extended masculinity-and-nation research beyond Euro-American contexts. Studies of nation-building and masculinity in India, postcolonial Africa, and contemporary East Asia show that masculine legitimacy and national belonging are co-produced under historically specific institutional conditions, even where the institutional forms differ substantially (Chakraborty, 2025; Ngoh, 2016; Liong and Chan, 2021; Hearn and Ratele, 2025). Cross-cultural portability of these claims remains an empirical question, not a prior assumption.

Recent developments have intensified these questions. Kimmel's (2013) account of aggrieved entitlement describes men who experience economic, demographic, or status displacement as people responding less to achieved dominance than to the perception of something lost. Ging's (2019) analysis of the manosphere identifies communities organized around grievance, victimhood, and aggrieved manhood rather than straightforward claims to dominance. Digital platforms amplify these formations and help them spread. Neither development displaces hegemonic masculinity as an analytic framework. Both reveal forms that persist, adapt, and propagate under conditions that earlier models did not fully anticipate.

The field now possesses sophisticated accounts of hegemony, adaptation, nationalism, militarization, grievance, and digital propagation. What remains less developed is a unified account of persistence itself. How do dominant masculine forms survive prolonged contestation? Why can support erode gradually and then destabilize rapidly? Why do some institutions continue to reproduce a dominant form while others abandon it? And how do dominant forms reconstitute themselves after displacement? The literatures reviewed above repeatedly approach these questions without fully resolving them. What the existing literature names—hegemony, adaptation, militarization, grievance, digital propagation—these works describe with great sophistication. What they do not yet specify is the underlying mechanics by which any of these forms is held in place across the heterogeneous fields that sustain it. Hegemonic masculinity theory tells us that dominance is contested and relational; it does not tell us why contestation rarely dislodges it. The hegemonic bloc tells us that dominant forms adapt by appropriation; it does not tell us why some appropriations stabilize and others fail. Gender-and-nation scholarship tells us that masculinity and nationhood are co-produced; it does not tell us why this co-production intensifies under specific conditions and fragments under others. What is missing across these accounts is a persistence mechanics — a specification of the recognitional and reinforcement dynamics through which dominant masculine forms remain operative even where they are widely repudiated, and through which they reorganize when repudiation crosses field-specific thresholds. The framework developed in §§3–4 supplies that specification, and §§5–8 trace its diagnostic and normative consequences.

## 3 Relational constitution: masculine configurations as recognition-dependent formations

Dominant masculine configurations are not traits that individual men possess. They are recognition-dependent social formations: arrangements of practices, dispositions, expectations, and entitlements that remain socially operative only insofar as they continue to be reproduced across interactional and institutional settings. This claim does not deny the importance of bodies, developmental histories, or material conditions. It makes a narrower point. The standards through which masculinity becomes socially intelligible persist only through ongoing social reproduction. When that reproduction weakens, masculine forms fragment, reorganize, or disappear.

A second distinction is essential. Masculinity names a broad symbolic-recursive domain: the historically variable field through which maleness becomes socially intelligible. Within that domain, particular forms emerge, compete, coexist, and sometimes become dominant. Among them is what this article terms the rigid configuration, organized around dominance, emotional invulnerability, hierarchy, disposability, and coercive legitimacy, and often described in public discourse under the contested label toxic masculinity. The domain itself is not reducible to any single form. The object of analysis is therefore not masculinity as such, but the conditions under which particular configurations persist, intensify, adapt, and decline.

The framework turns on a distinction that is frequently obscured in public discussion. Recognition does not mean approval. It refers to the practical reproduction of a configuration through ongoing social uptake. A masculine form is recognized when peers, families, workplaces, media systems, and state institutions continue treating it as an operative standard of what a man is and ought to be. Approval, legitimacy, standing, and status may accompany recognition, but they are analytically distinct. A configuration may command low approval, possess contested legitimacy, and still remain socially operative. Recognition can be enthusiastic, strategic, habitual, resentful, or coerced. Men who privately reject a dominant form may nevertheless reproduce it through silence, deference, avoidance of ridicule, or anticipatory self-regulation. Recognition-dependence therefore predicts something counterintuitive: a masculine form can remain highly operative in environments where it is widely disliked. Recognition in the present framework does not adopt the normative-developmental sense developed by Honneth (1995), for whom intersubjective recognition names the condition required for subjects to achieve a fully realized self-relation. The concern here is narrower and structural: not the normative grounds on which recognition ought to be extended, but the social conditions under which particular configurations remain operative as social standards.

This is why the relevant unit of analysis is the recognitional field rather than the individual attitude—a structured site of social reproduction through which a configuration is sustained as an operative standard by the organized uptake of those positioned within it. Masculine configurations persist through organized sites of reproduction: peer groups, workplaces, labor markets, families, media systems, digital platforms, and state institutions. These fields differ in their gatekeepers, reward structures, and modes of uptake. They are only loosely coordinated. A configuration may therefore confront individuals as an external constraint they did not choose while remaining dependent, at the field level, on continued reproduction. In this respect, the framework extends broader social-ontological approaches that treat socially constituted phenomena as both real and dependent on collective uptake (Goffman, 1959; Searle, 1995; Haslanger, 2012; Epstein, 2015). The additional claim made here is temporal: persistence itself requires continuing reproduction across multiple sites (Kidwell, forthcoming).

The account is best understood as a model of contested persistence, not smooth reproduction. Connell and Messerschmidt (2005) explicitly reject any reading of hegemonic masculinity as a straightforward model of social reproduction. Dominant forms persist through struggle, contradiction, adaptation, and uneven reproduction. Demetriou's (2001) concept of the hegemonic bloc provides the clearest example. Dominant forms remain dominant partly by incorporating elements of subordinate and oppositional masculinities and reworking them into new arrangements. Adaptation is therefore not the opposite of persistence. It is one of persistence's principal mechanisms. Visible change in style, language, or presentation may coexist with substantial continuity in social position.

Two properties of recognition-dependent persistence shape the remainder of the argument. The first is threshold sensitivity. Support for a configuration can erode gradually while producing little visible change, then destabilize rapidly once reproduction falls below a critical level. The second is field specificity. Configurations do not disappear all at once. Reproduction is withdrawn field by field. A masculine form may lose legitimacy in professional, educational, and therapeutic settings while remaining robust in peer cultures, entertainment industries, or digital communities. What often appears as social inconsistency is frequently the ordinary consequence of uneven reproduction across different fields.

This perspective also clarifies a pattern commonly interpreted as personal hypocrisy. A person may sincerely criticize a masculine configuration in political or discursive settings and, later the same day, participate in its commercial reproduction through the entertainment they consume, share, or enjoy. The framework treats these actions as different forms of participation operating in different fields. Discursive opposition is real. Commercial participation is real. Neither cancels the other. The paradox disappears once recognition is distinguished from approval. People routinely reproduce configurations they do not endorse because participation and endorsement are different social relations. The persistence of a configuration depends on the former more than the latter.

The argument developed here remains descriptive. It identifies the conditions under which masculine configurations persist, adapt, fragment, and reorganize. Questions of harm, exclusion, and alternative arrangements are taken up later. What this section establishes is the ontological foundation of the framework: dominant masculine configurations are recognition-dependent formations whose persistence is contested, field-specific, and sustained through ongoing social reproduction rather than simple endorsement.

## 4 Recursive stabilization: habitus, field convergence, and path dependence

The preceding section argued that masculine configurations persist through reproduction across recognitional fields rather than through intrinsic stability. A further question follows: why do men embedded within dominant forms so often experience them as binding, and why do critique, education, and normative pressure frequently fail to dislodge them? A common answer emphasizes psychological internalization. The framework developed here points elsewhere. Rigidity emerges less from what individuals believe than from the convergence of multiple fields reinforcing the same practices and from the embodied dispositions those fields cultivate. The result is a form of stability that is distributed, relational, and resistant to isolated intervention.

The most developed sociological account of this stabilization is Bourdieu's concept of habitus: the durable, embodied system of dispositions formed through extended exposure to the conditions of a field, and continuously re-fitted to that field through practical engagement with it (Bourdieu, 1977, 1990). Habitus is not a set of beliefs but a feel for the game—a practical grasp of how to act, react, and present oneself within a given setting, acquired pre-reflectively and largely below the level of explicit articulation. Applied to masculinity, habitus designates the embodied disposition through which a man learns, without explicit deliberation, how to occupy his body in male company, how to pitch a voice when stakes rise, when to escalate and when to retreat, what to find funny and what to find shameful, and which postures register as competent. This is the level at which dominant masculine configurations are most securely held. Critique aimed at conscious belief leaves habitus intact, because habitus does not operate at the level of belief.

The rigidity of any particular masculine configuration, however, is not produced by habitus alone. A disposition formed through exposure to one field would, on its own, be revisable through exposure to differently structured fields. What makes a dominant configuration resistant to revision is field convergence of multiple fields rewarding the same performance simultaneously. Peer interaction, workplace evaluation, romantic and sexual markets, family expectations, entertainment and media representation, and—for the configurations examined later in the article—the institutions of the nation-state often constitute parallel reinforcement streams, each operating by its own logic but pulling in compatible directions. A man whose disposition has been formed across several such fields confronts a redundant source of feedback rather than a single one: deviation in any one field is met by penalty, and conformity is rewarded from multiple directions at once. It is this field convergence, not depth of internalization, that produces the patterns of rigidity masculinity scholarship has long described.

This conception reframes the costs of deviation in social rather than psychological terms. The familiar observation that alternative masculine practices—vulnerability, emotional disclosure, the refusal of certain hierarchies—feel impossible to those embedded in a rigid configuration is, on the present account, less a claim about cognitive limits than about field gradients. Alternatives remain conceivable but are interactionally penalized: a man who deviates does not fail to imagine an alternative practice, he encounters—in the form of ridicule, withdrawn approval, status reassignment, and lost standing—the practical reasons why the field he inhabits does not support it. To describe such alternatives as becoming socially unintelligible is to describe a feature of the field, not a limitation of the actor: the field's response gradient renders certain practices illegible as competent masculine conduct. This formulation preserves agency. Persistence does not require that anyone be fooled or impaired; it requires only that the price of deviation remain higher than the price of conformity across the fields that constitute the actor's recognitional environment.

Configurations sustained by convergent reinforcement also exhibit characteristic dynamics under perturbation. Sociological work on threshold behavior (Granovetter, 1978; Schelling, 1978) has long established that individual willingness to adopt or withdraw from a given practice typically varies across actors and is sensitive to the proportion of others observed to be doing so. Where individual thresholds are distributed across a population, field-level shifts proceed as cascades: a configuration that has held for an extended period can destabilize rapidly once enough actors cross their individual thresholds in close succession. Subsequent network-theoretic work has refined this picture in two directions relevant to the analysis to follow.

Local reinforcement—the patterned reward and sanction occurring within tightly coupled fields such as peer groups, workplaces, or family systems—produces threshold behavior of the classical type, in which cascades are constrained by the structure of immediate interaction. Network amplification operates differently: under certain network conditions, signals propagate widely through structurally distinct pathways, and behaviors requiring multiple sources of confirmation spread along routes unavailable to face-to-face fields (Watts, 2002; Centola, 2010). Peer and workplace fields operate predominantly through local reinforcement. Digital infrastructures alter masculine reproduction through changes in amplification topology. The infrastructures examined in §6 diverge largely along this dimension. What both modes share is that they are dynamics of coordination, not of psychological lock-in: thresholds are properties of populations within fields, not measures of any individual's depth of commitment. The terms threshold, cascade, attractor, and hysteresis are used throughout what follows as qualitative characterizations of social dynamics—descriptive analogies drawn from the complexity and network literatures—rather than as formal mathematical models.

A final feature of recursive stabilization warrants explicit attention. Configurations sustained by convergent field reinforcement do not, in general, revert symmetrically when the conditions producing them weaken. The dispositions formed through long exposure to a field and the field structures themselves both accumulate, with the result that the conditions required to dismantle an established configuration are typically more demanding than those that initially established it. Bourdieu's later work describes this as the hysteresis of habitus: the persistence of dispositions formed under prior field conditions, often beyond the conditions that produced them (Bourdieu, 1990). For the purposes of the present analysis, the implication is methodological rather than metaphorical. A configuration reinforced across several fields for an extended period will not dissolve simply because reinforcement in one field is withdrawn; both the dispositions formed under prior conditions and the structuring effect of slowly reorganizing fields persist. Persistence carries a temporal asymmetry: dominant masculine configurations are easier to maintain than to dislodge, and the work of dislodging them involves not the reversal of past reinforcement but the construction of differently structured fields under present ones.

Three implications follow. First, accounts of contemporary masculinity that locate rigidity in the depth of individual conviction systematically underestimate the role of field convergence; the appropriate object of explanation is the structure of the reinforcement environment, not the psychology of the actors moving through it. Second, threshold dynamics and path dependence explain why dominant forms can appear stable for long periods, destabilize rapidly, and later reconstitute through partial fields and digital infrastructures rather than reverting symmetrically. Third—and most consequential for the normative argument developed in §8—critique alone is rarely sufficient: dislodging an established configuration requires the construction of alternative fields capable of conferring recognition on alternative dispositions, not the persuasion of individuals already shaped by the convergence of fields the configuration depends on. The next section turns from these stabilization mechanics to what is specifically injured under such configurations, and to the positional and relational harms that result.

## 5 Positional legitimacy and humiliation: the harm mechanism

The framework developed to this point is descriptive: it specifies the conditions under which masculine configurations persist, fragment, and reorganize without yet asking what is wrong with any particular configuration or what makes change worth pursuing. The present section relaxes that descriptive restraint along a carefully bounded axis. It introduces the question of positional injury—what is harmed, and to whom, when a configuration of the kind analyzed in §§3–4 is sustained—and identifies the mechanism through which the dynamics examined so far become harms in a sociologically robust sense rather than ordinary features of social organization.

The methodological commitment governing what follows must be stated explicitly. An account of why a configuration persists is not a justification of it; the analysis offered here explains persistence but does not excuse harm. To trace dominance behaviors to conditions of positional precarity is not to soften their consequences for those they target, nor to transfer responsibility from the actors who enact them to the conditions that incentivize them. The framework treats persistence as a sociological problem and harm as a moral one, and maintains that the two questions remain analytically distinct even where their answers become entangled. What follows is an account of mechanism, not a mitigation of injury.

Sociological treatment of harm in recognition-dependent systems is sharpened by Lawson's (2022) account of social positioning, developed within Cambridge social ontology. On Lawson's view, the basic unit of social organization is not the individual practice but the position: a structured location within a community to which are attached rights, obligations, powers, and standing—where standing designates the recognitional grounds on which a position's rights and obligations count as legitimate within a given field—the practical currency of social legibility. Persons and other phenomena become socially organized through processes by which positions are constituted and through which individuals are incorporated into them. Within this framework, the masculine configurations analyzed in §§3–4 are distributions of social position: standards by which men are sorted into legible categories—competent or weak, respected or disposable, audible or invisible—to which differential rights, obligations, and entitlements are attached. To occupy a recognized masculine position is to be situated within a structured allocation of standing; to fail at occupying one is to lose access to the recognitional grounds on which that standing depends.

This positional framing clarifies a feature of rigid masculine configurations that purely behavioral accounts often struggle to explain. Dominance signaling, hierarchy enforcement, and aggressive performance are frequently most pronounced under conditions of positional precarity rather than secure dominance. Where substantive position is materially or institutionally available through labor, occupational standing, relational embeddedness, or civic participation, symbolic compensation tends to be less necessary. Where such position is unavailable or eroding, symbolic mechanisms of elevation become correspondingly more salient. Aggression, status performance, intimidation, ideological rigidity, and the assertion of hierarchy can produce localized recognitional gains within fields configured to reward them. Such gains are artificial only in the sense that they do not track substantive standing; the recognition itself is socially real within the field that confers it, and the relief it provides from positional collapse is correspondingly real. Domination, on this account, is frequently downstream of recognitional dependence. That is a sociological observation about mechanism, not an evaluative claim about responsibility.

Masculinity scholarship has repeatedly noted the disproportionate intensity with which humiliation, ridicule, emasculation, and public exclusion are met within rigid masculine configurations, but the mechanism remains underdeveloped. Humiliation, on the present framework, is not primarily an affective injury but a positional injury. To be humiliated within a masculine field is to be visibly removed from a recognized position—to be stripped, before witnesses whose recognition was constitutive of that position, of the standing on which masculine legibility rested. Because the position is recognition-dependent, the removal is interactional rather than internal. It occurs in the field's response gradient rather than in the actor's self-assessment. The intensity of the response to humiliation tracks not the depth of an individual's vulnerability but the centrality of positional standing to the configuration. This perspective also clarifies why humiliation has repeatedly functioned as an engine of nationalist political projects. Enloe's (1989) observation that nationalism feeds on masculinized memory, masculinized humiliation, and masculinized hope becomes more precise in these terms. Humiliation understood as positional injury is converted, through nationalist mobilization, into a project of recognitional reanchoring at the scale of the state, in which the recovery of masculine standing is offered as the recovery of national standing.

Treating masculine configurations as positional systems sustained through distributed recognition aligns with and extends the conception of power developed in Foucault's later work. Power, on Foucault's account, is a productive, capillary, and relational force that produces subjects rather than simply constraining them, distinct from sovereign power exercised downward from a single source (Foucault, 1990, 1995). Masculine subjects, on the present framework, are produced through recognition-power dynamics distributed across fields. The configurations analyzed in §§3–4 do not merely regulate pre-existing male individuals; they constitute the socially legible positions through which masculine subjectivity becomes available in the first place. This perspective clarifies a feature of rigid masculine configurations that the rhetoric of domination often obscures. The harms they produce are reproduced across actors who would individually disclaim them because the mechanism of reproduction is field-level uptake rather than sovereign decision. That distinction carries consequences for both diagnosis and intervention.

Two clarifications should close this section. First, the positional dynamics analyzed here injure those positioned as the targets of masculine domination: women, subordinated and marginalized masculinities, queer subjects, and those rendered disposable by the hierarchies these configurations enforce. These are the principal harms such configurations produce, and they are not mitigated by the additional observation that the same configurations may also impose costs on the men embedded within them. The two harms are not symmetric, and explanation of the second does nothing to soften the first. Second, the framework does not relocate responsibility from agents to conditions. Conditions structure the field; agents act within it, including the agents who enforce configurations, exclude those who deviate from them, and benefit from the positional rewards they distribute. The configurations analyzed here produce injury through humiliation, exclusion, emotional restriction, coercive positional enforcement, recursive alienation, and symbolic disposability. Those injuries are sustained through the recognitional infrastructures by which fields confer or withhold standing, not through individual ill will alone. The next section turns to the specific infrastructures through which these dynamics operate.

## 6 Recognitional infrastructures of masculine reproduction

The framework developed across the preceding sections specifies a general mechanism: masculine configurations persist through recognition-dependent reproduction stabilized by field convergence and shaped by positional dynamics. The present section turns from mechanism to application. It examines four contemporary infrastructures through which masculine configurations are reproduced—labor and economic precarity, militarism, nationalist political projects, and digital communication—and argues that each operates through a distinct reproductive logic. These are defined here as compensatory standing infrastructures, sacrificial legitimacy infrastructures, collective recognitional reanchoring, and amplification infrastructure. The cases that follow are illustrative rather than evidentiary. No claim is made to empirical demonstration of any particular domain. The purpose is narrower: to show how the persistence apparatus developed in the preceding sections accounts for differences among infrastructures that are often treated as variations of the same phenomenon, and how those differences sharpen sociological analysis within the masculinity-and-nation literature. The four infrastructures are summarized in Table 1.

**Table 1:** Four recognitional infrastructures of masculine reproduction.

Infrastructure	Standing source	Primary field	Reproductive logic
§6.1 Compensatory	Substitute symbolic status	Labor markets, peer networks	Confers standing where substantive position has eroded
§6.2 Sacrificial	Service and expendability	Military institutions, state	Legitimacy granted through willingness to sacrifice
§6.3 Reanchoring	Collective restoration	Nationalist politics, state	Recovers masculine standing at the scale of the nation
§6.4 Amplifying	Visibility and network confirmation	Digital platforms, algorithmic curation	Scales locally bounded configurations through network topology

### 6.1 Labor precarity: compensatory standing infrastructure

Sociological work on masculinity and economic life has long observed that men experiencing labor precarity, deindustrialization, and downward mobility—the closure of manufacturing sectors, the contraction of stable wage employment, and the gradual erosion of the provider role—often display intensified commitment to rigid masculine configurations. Kimmel's (2013) account of aggrieved entitlement names the affective and political formation through which men experiencing displacement come to feel that something rightfully theirs has been taken, and through which that sense of loss becomes organized into contemporary restorationist masculine politics. The framework developed here specifies the mechanism. Traditional masculine positioning was anchored in labor competence, the provider role, occupational standing, civic embeddedness, and institutional respect. These recognitional fields conferred standing through materially grounded participation. When those fields erode, the dispositions formed within them persist while the standing they once conferred becomes less available. The resulting gap is asymmetrical. Substantive position has been withdrawn, but the expectations formed around it remain.

Under such conditions, compensatory standing infrastructures emerge. These are fields that confer symbolic position where substantive position has become scarce. Local hierarchies of dominance signaling, restorationist political movements, hyper-competitive peer environments, and reactionary cultural formations operate in this register. They function as substitute infrastructures, supplying forms of standing that declining labor and civic institutions no longer reliably provide. Such gains are real within the fields that confer them, and the relief they offer from positional collapse is correspondingly real. The framework therefore locates domination less in secure superiority than in attempts to recover standing under conditions of erosion and uncertainty.

This account does not soften the consequences of compensatory standing. Where standing is acquired through domination, it is acquired at the expense of others. The standing of subordinated actors becomes the resource from which compensatory standing is extracted. The mechanism explains how such arrangements persist; it does not lessen the harms they produce.

### 6.2 Militarism: sacrificial legitimacy infrastructure

Militarized masculinity operates through a different logic. Where the infrastructures discussed in §6.1 supply symbolic standing under conditions of erosion and uncertainty, militarism confers standing through the expenditure of self. The state institutionalizes a configuration in which masculine legitimacy is granted on condition of willingness to sacrifice—bodily, laboring, and ultimately existentially—for a collective purpose. Conscription regimes, veteran-honor practices, and martial commemoration function as positional ladders through which standing is allocated. The legitimacy of the configuration is grounded through sacrifice itself rather than despite it.

Enloe's (1989, 2014) analysis of militarized masculinity traces this infrastructure across the gendered political economy of bases, borders, and conscription. Nagel (1998) shows how sexualized militarism extends these dynamics beyond formal military institutions through the construction of enemy figures, the policing of the home front, and the symbolic authority of soldierly icons. The result is a configuration whose reach extends well beyond armed service, shaping broader understandings of citizenship, duty, and belonging.

Within the present framework, sacrificial legitimacy infrastructures are distinguished by the linkage they establish between standing and expendability. The same configuration that honors service also constrains those who perform it. Expectations surrounding emotional expression, endurance, pain, vulnerability, and refusal are built into the very criteria through which standing is conferred. Those who cannot or do not enter the infrastructure are often positioned as deficient or insufficiently masculine, while those outside its protective boundaries face the consequences of the legitimacy it generates. Civilians designated as enemy populations, communities rendered expendable, and those subjected to militarized projects bear the principal harms sustained by the standing such configurations confer.

### 6.3 Nationalism: collective reanchoring infrastructure

Where compensatory infrastructures supply standing locally and sacrificial infrastructures confer it through service, nationalist masculine politics operates through collective recognitional reanchoring: the promise of recovered masculine standing at the scale of the nation-state under conditions of widespread destabilization. The long-standing observation that manhood and nationhood are co-produced (Nagel, 1998) can be specified more precisely within the present framework. Nationalist projects function as recognitional infrastructures when masculine standing becomes uncertain across multiple domains of social life. Labor, family, and civic institutions may no longer provide stable grounds for recognition, but nationalist mobilization offers an alternative route through which standing can be restored collectively by linking masculine legitimacy to the symbolic recovery of national legitimacy.

Yuval-Davis's (1997) account of gendered citizenship identifies men as defenders and political agents of the nation, while women are positioned as its biological, cultural, and symbolic reproducers. Nationalist projects activate and intensify these allocations under conditions of broad destabilization. The process becomes especially powerful when dispersed forms of compensatory standing no longer provide sufficient recognitional support. What appears politically as a project of national renewal simultaneously functions as a project of masculine restoration.

The Enloe formulation introduced in §5—that nationalism feeds on masculinized memory, masculinized humiliation, and masculinized hope—captures the affective surface of this process. The mechanism operates through the conversion of humiliation experienced as positional injury into a collective political project. Individual experiences of lost standing are translated into narratives of national decline, and the recovery of masculine standing is presented as inseparable from the recovery of national standing. This helps explain why “traditional values” politics, narratives of national humiliation, and the restoration of patriotic masculine icons intensify under conditions of broad destabilization rather than secure dominance. These projects frequently draw upon the same grievances that animate the infrastructures discussed in §6.1, but they operate through a different logic. Compensatory standing acts locally. Collective recognitional reanchoring acts at the scale of the nation and mobilizes the institutional capacities of the state in ways local infrastructures cannot.

### 6.4 Digital infrastructures: amplification

The fourth infrastructure differs from the preceding three in the mechanism through which reproduction occurs. The distinction introduced in §4 between local reinforcement and network amplification becomes central here. Peer groups, workplaces, and family systems reproduce masculine configurations through tightly coupled local interactions in which reinforcement is constrained by immediate social relationships. Digital communication infrastructures operate differently. Visibility, exposure, and confirmation are shaped by algorithmic systems rather than face-to-face interaction, allowing patterns of behavior that require repeated social confirmation to spread through pathways unavailable to local fields (Watts, 2002; Centola, 2010).

Ging's (2019) analysis of the manosphere illustrates this dynamic. Communities organized around identities such as incel, “beta,” redpill, and related formations complicate conventional assumptions that masculine power is always associated with secure dominance. Many of these communities instead organize around narratives of victimhood, exclusion, and aggrieved manhood. The communicative affordances of digital media are especially effective at amplifying such formations because they allow geographically dispersed participants to converge within a shared recognitional environment.

Subsequent research has documented an increasingly interconnected manosphere and shown how recommendation systems, cross-platform migration, and algorithmic curation contribute to the diffusion of misogynistic and grievance-centered content (Ribeiro et al., 2020; Mamié et al., 2021; Baker et al., 2024). What has emerged is less a single community than an evolving network of partially overlapping groups linked by shared grievance and anti-feminist narrative, with digital platforms shaping not only what circulates but the pathways through which participants move between communities.

Within the present framework, the manosphere functions as an amplification infrastructure. It provides an alternative environment of recognition for men experiencing institutional displacement, combining the compensatory dynamics described in §6.1 with forms of propagation distinctive to digital media. Recommendation systems, content pipelines that connect entry-level grievance material to progressively more rigid forms, and the algorithmic curation of engagement systematically reward certainty, outrage, grievance, and hierarchy conflict. The result is a field increasingly oriented toward high-intensity signaling. Under sustained amplification, arrangements stabilized through local reinforcement and amplified visibility can exhibit attractor-like dynamics, intensifying recursively toward forms whose persistence owes as much to the structure of amplification as to underlying social demand.

The infrastructure does not generate the underlying recognitional dependence. That dependence is inherited from the compensatory and sacrificial dynamics discussed in §§6.1–6.2. What digital infrastructures contribute is a propagation environment in which forms that might otherwise remain locally bounded can scale rapidly, separate from mainstream fields, and reconstitute themselves as durable alternative recognitional formations.

## 7 Reframing the “crisis of masculinity”

The diagnosis that contemporary societies are experiencing a “crisis of masculinity” appears regularly in both popular and academic discourse. The diagnosis captures something real, but it also conflates two analytically distinct phenomena. The framework developed here requires those phenomena to be separated. Under contemporary conditions, the crisis is not masculinity as such. Masculinity remains an actively reproduced symbolic-recursive domain across virtually all of the infrastructures examined in §6. The crisis concerns particular hegemonic configurations whose recognitional support has eroded unevenly across the fields that once sustained them. Restated in the vocabulary of this framework, the substance of crisis discourse is recognitional destabilization: weakening uptake, declining intelligibility, and contested legitimacy within fields whose prior alignment had made a configuration appear natural, stable, and dominant. Any attempt to move beyond reductive crisis narratives must begin with that distinction.

Destabilization does not produce collapse. It produces fragmentation and reorganization. Recognitional withdrawal proceeds unevenly across fields, each operating according to its own thresholds, timelines, and conditions of reproduction. Configurations that lose legitimacy in professional, educational, or therapeutic settings may remain robust within peer networks, commercial environments, sacrificial institutions, or digital communities. Distinct forms therefore come to coexist within the same society, sustained through partially distinct recognitional fields. The literature on hegemonic masculinity has described aspects of this pattern in related vocabularies. Demetriou's (2001) hegemonic bloc identifies hybridization as a mechanism of adaptation rather than dissolution, while Connell and Messerschmidt's (2005) reformulation emphasizes the multi-scalar relations through which local, regional, and global masculinities are sustained. The framework developed here adds a further implication. Under conditions of recognitional destabilization, rigid configurations may intensify rather than recede because they continue to supply certainty, intelligibility, and standing at precisely the moment alternative infrastructures become less reliable. Restorationist politics, manosphere formations, and the resurgence of “traditional values” projects are therefore better understood as consequences of destabilization than as evidence against it.

The resulting diagnosis differs from conventional crisis narratives. What is often described as a crisis of masculinity is more accurately understood as a reorganization of masculine recognitional fields under conditions of economic, institutional, and digital change. Configurations displaced from mainstream fields do not simply disappear. They reconstitute themselves through alternative infrastructures such as those examined in §§6.1 and 6.4, emerging as adapted forms sustained through different reinforcement and amplification dynamics. This reframing also clarifies the relationship between “masculinity in crisis” and resurgent nationalism. The two are not opposing conditions. They emerge from the same processes of destabilization and reorganization. Responses directed solely at particular configurations will therefore remain incomplete. The question that follows is how alternative recognitional infrastructures might be constructed and sustained.

## 8 Healthier masculinities and alternative recognition structures

What the preceding sections establish is a constraint on any account of change. Configurations sustained through field convergence, positional dynamics, and amplification processes cannot be dissolved through critique alone because their persistence does not reduce to belief. Critique operates within discursive fields. The recognitional infrastructures examined in §6 operate across peer, occupational, sacrificial, national, commercial, and digital environments whose reproductive logics are often largely unaffected by the discursive rejection of any single form. This is not a counsel of futility. It is a consequence of the mechanism described throughout the article. Where a configuration is sustained by infrastructures operating according to their own logic, intervention must engage those infrastructures as well as the forms they reproduce.

The framework also rules out a second conclusion: that masculine recognition can be dismantled wholesale. Recognition-dependent positioning is not a contingent feature of patriarchal arrangements but a general feature of social positioning itself. Masculinity, as a symbolic-recursive domain through which maleness becomes socially legible, is reproduced through those processes whether particular forms are celebrated or repudiated. Attempts to withdraw recognition from masculinity as such leave the underlying demand for standing intact while narrowing the channels through which standing can be conferred. Under the conditions described in §6, the likely result is not dissolution but reconstitution through the infrastructures least dependent on mainstream institutional legitimacy. Healthier masculine forms therefore confer standing on different grounds; they do not eliminate the demand for standing altogether. They must remain interactionally legible as legitimate masculine practice within the positional grammar of the fields that confer recognition.

A related phenomenon becomes visible when the analysis is brought down to the level of everyday interaction. Many male peer groups already provide care, affirmation, support, and belonging, but they often do so through masculine-coded symbolic practices rather than through the language conventionally associated with emotional expression. Competitive affirmations such as you crushed it, you stepped up, and you made it look easy, together with ritual teasing, sustained presence during hardship, handshakes, shoulder grips, and the ordinary reliability of friendship, function as recognition technologies—micro-practices through which standing is conferred and belonging reproduced within peer fields. They are not failed versions of emotional expression awaiting correction. They are existing mechanisms through which recognition is distributed. At the scale of friendships, families, work crews, and teams, they illustrate how masculine standing can be organized around reliability, competence, presence, and reciprocity rather than dominance, invulnerability, or hierarchy. The broader implication is structural. Healthier masculine forms are most likely to emerge through the redirection of existing recognition technologies toward different criteria of standing rather than through the replacement of masculine recognition itself.

The conditions for change therefore lie in the construction of alternative recognitional infrastructures. These would be fields and institutions organized around reciprocity, durable relational competence, restraint, civic embeddedness, and care, while remaining capable of conferring standing in ways that are recognizable as legitimate masculine practice. The framework does not determine the design of such infrastructures. That is an empirical and political question whose answer depends on particular institutions, histories, and societies. What the framework can specify are the conditions such infrastructures must satisfy. They must confer recognition durably across relevant fields, distribute standing through criteria distinct from those of the forms they seek to replace, and operate at sufficient scale to compete with the compensatory and digital infrastructures that currently absorb displaced demands for standing. None of this amounts to a program. It identifies the conditions under which partial, contested, and uneven change becomes possible and clarifies why the concerns reflected in Sustainable Development Goals 5 and 10 ultimately require attention not only to harmful configurations themselves but also to the infrastructures through which they are reproduced.

## 9 Scope, boundaries, and anticipated objections

This section makes explicit several boundaries that have guided the argument throughout. Doing so serves two purposes. It helps pre-empt predictable misreadings and clarifies the limits the framework places on its own claims.

One possible boundary concerns normative evaluation. Explaining why rigid configurations are experienced as binding is not the same as excusing the harms they produce. The discussion in §5 made explicit who bears the principal burdens of masculine domination. The framework's object of analysis is the persistence of dominant masculine configurations in general. The rigid arrangement often described through the contested public label toxic masculinity is only one historically and culturally specific subtype of that broader phenomenon. Understanding the mechanisms through which such forms persist neither mitigates nor justifies their consequences. It clarifies the conditions under which they endure.

Another possible objection is that the framework represents an idiosyncratic system imported into the sociology of masculinity from outside the field. That characterization would overstate its novelty. The only proprietary apparatus introduced is the relational-constitution framework cited as forthcoming, itself a sociology-facing social-ontological project. The remaining theoretical resources are well established within the conversations this article enters: hegemonic masculinity, habitus, social positioning, Foucault's account of productive power, the threshold and complex-contagion literatures, and the gender-and-nation tradition associated with Nagel, Enloe, and Yuval-Davis. The contribution claimed here is therefore one of synthesis and application. The framework assembles these resources into a unified account of persistence that operates beneath existing explanations rather than replacing them.

A more substantial objection concerns explanatory scope. An account organized around recognition may appear to elevate recognitional dynamics above material, coercive, institutional, and economic determinants of masculine reproduction. The framework rejects that implication. Recognition-dependent persistence is a claim about the conditions under which social forms remain operative, not a claim about the sole basis of masculine practice. Economic conditions, labor-market structures, material deprivation, coercion, state violence, institutional capacities, and the distribution of resources retain independent causal force throughout the analysis. The framework treats such factors as part of the environment within which recognitional infrastructures emerge, stabilize, and change. The relationship is reciprocal. Labor precarity reshapes the infrastructures examined in §6.1. State institutions help sustain the legitimacy structures examined in §6.2. The framework is most useful where it clarifies how material conditions and recognitional processes interact. It reduces nothing to recognition. It identifies what recognition contributes to the explanation.

Two additional objections can be addressed together. The first concerns determinism. Terms such as cascades, hysteresis, reinforcement, and amplification may suggest a theory of social lock-in. That is not the claim being made. As §4 argued, the mechanism operates through probabilities, tendencies, and shifting costs rather than inevitabilities. Actors remain capable of recognizing alternatives even when the costs of acting on them differ substantially across fields. The second objection concerns the absence of original empirical data. This article is a Hypothesis and Theory contribution. The cases discussed in §6 are illustrative rather than evidentiary. Their purpose is to demonstrate the framework's analytic reach and to identify questions for future empirical investigation.

## 10 Empirical predictions and operationalization

The framework generates a set of distinguishable empirical predictions. Each identifies observable patterns that should emerge if the account developed here is correct, and each provides points of comparison with adjacent explanations of masculine reproduction and change.

1) Field-specific dissolution. The framework predicts that the repudiation of a masculine configuration will proceed unevenly across fields. Discursive fields, including educational, professional, therapeutic, and journalistic environments, should exhibit measurable disavowal earlier than reinforcement fields such as commercial entertainment, peer subcultures, and digital communities. Existing accounts of hegemonic masculinity, treating dominance as a more unified social position, offer no equally specific prediction about the timing or distribution of decline. Longitudinal comparison across field types using content analysis, ethnography, and platform data would provide a direct test.

2) Threshold nonlinearity. Recognitional support may erode for extended periods with little observable change in behavioral reproduction before destabilization occurs rapidly once field-specific thresholds are crossed. Gradualist accounts predict proportional change throughout the process. Longitudinal measurement of attitudes and behavior within identifiable populations would distinguish these competing expectations.

3) Compensatory intensification under precarity. Rigid masculine forms should intensify most visibly among populations experiencing labor-market collapse, status displacement, or institutional withdrawal, controlling for baseline levels. Comparative survey and ethnographic research across deindustrialized regions, post-state-socialist transitions, and economically stable labor markets would provide a clear test.

4) Complex contagion within amplification infrastructures. Configurations propagated through digital environments should exhibit network-contagion dynamics, particularly patterns requiring multiple reinforcing exposures, that differ from forms sustained primarily through local reinforcement. Digital trace data, propagation analysis, and comparison of online and offline adoption trajectories would allow these dynamics to be measured directly.

5) Asymmetric critique failure. Critique operating predominantly within discursive fields should produce measurable attitudinal change without corresponding behavioral change in fields sustained through non-discursive reinforcement. Major public critique campaigns provide natural opportunities for comparing shifts in expressed belief with shifts in observable practice. The framework predicts the former to outpace the latter.

6) Infrastructure convergence. Intensification should be greatest where multiple recognitional infrastructures converge on the same population. Populations simultaneously exposed to compensatory, sacrificial, reanchoring, and amplifying dynamics should display stronger and more durable forms of masculine reproduction than populations influenced by any single infrastructure alone. Cross-national comparison of contemporary nationalist masculine projects would provide an especially useful testing ground.

Taken together, these predictions constitute the empirical purchase of the framework. They are distinguishable, operationalizable, and comparable across contexts. Their value lies not in guaranteeing confirmation but in making disagreement empirically tractable and in providing a research agenda for the comparative and transnational sociology of masculinity and nationhood.

## 11 Conclusion

This article has proposed a persistence mechanics for hegemonic masculine configurations under contemporary social, economic, and digital conditions. Its central claim is straightforward: dominant masculine forms persist not through intrinsic psychological or cultural stability but through coupled recognitional and reinforcement dynamics distributed across heterogeneous infrastructures. Those infrastructures operate through distinct logics: compensatory standing, sacrificial legitimacy, collective recognitional reanchoring, and amplification. The contribution is not to displace these accounts but to specify what they leave underspecified: why contestation so rarely dislodges dominant configurations, why destabilization proceeds asymmetrically across fields, and how reconstitution occurs through partial infrastructures rather than simple reversion. Hegemonic masculinity theory explains what dominance is; the hegemonic bloc explains how it adapts; Bourdieu explains how dispositions persist. What this framework adds is the mechanics by which configurations survive, fracture, and reorganize across heterogeneous fields operating by analytically distinct logics.

Two implications follow. First, critique directed at the beliefs of those embedded within rigid configurations rarely dissolves them because the mechanism of persistence does not reduce to belief. Change requires attention to the infrastructures through which forms are reproduced, not only to the arrangements those forms sustain. Second, what is commonly described as a crisis of masculinity is more accurately understood as the recognitional destabilization of particular hegemonic forms and the reorganization of the infrastructures that previously sustained them. Restorationist politics, manosphere amplification, and nationalist masculine projects appear, in this account, as predictable consequences of destabilization within recognition-dependent fields rather than as separate or opposing developments. Where healthier masculine forms emerge, they do so through alternative recognitional infrastructures capable of making different grounds of standing interactionally recognizable as competent masculine practice. The task is not the abolition of masculine legitimacy but its reorganization around different criteria.

The framework is offered as conceptual infrastructure for empirical inquiry rather than as a closed theoretical system. The conditions highlighted by this Research Topic, including militarism and veteranhood, labor precarity, nationalist mobilization, digital cultures, and the gendering of citizenship and belonging, provide especially fertile terrain for testing its claims. Under what institutional and economic conditions do compensatory standing infrastructures intensify? How do sacrificial legitimacy and collective reanchoring interact within particular national projects? What enables alternative recognitional infrastructures to acquire the scale and amplification necessary to compete with established forms? These questions are ultimately empirical. The value of the framework will depend on whether it helps make visible dynamics that existing accounts have left unexplained or only partially understood.
